# The Association between Food Insecurity and Making Hunger-Coping Trade-Offs during the COVID-19 Pandemic: The Role of Sources of Food and Easiness in Food Access

**DOI:** 10.3390/nu14214616

**Published:** 2022-11-02

**Authors:** Ye Luo, Catherine Mobley, Leslie Hossfeld, Caitlin Koob, Cassius Hossfeld, Samuel L. K. Baxter, Sarah F. Griffin

**Affiliations:** 1 Department of Sociology, Anthropology and Criminal Justice, Clemson University, Clemson, SC 29634, USA; 2 College of Social, Behavioral and Health Sciences, Clemson University, Clemson, SC 29634, USA; 3 Department of Public Health Sciences, Clemson University, Clemson, SC 29634, USA

**Keywords:** COVID-19, food access, food insecurity, nutritional insecurity, rural residents, trade-offs

## Abstract

Many individuals make financial, health and food related trade-offs to cope with the challenges of food insecurity and to meet their household needs for healthy, affordable food. A survey (*n* = 652) was conducted in nine rural counties in South Carolina, USA, during the COVID-19 pandemic from August 2020 to July 2021. We examine if level of food insecurity predicts hunger-coping trade-offs, and whether this relationship is moderated by easiness in food access and dependence on different food source types. Nearly one-third of the respondents experienced food insecurity. Making trade-offs between paying for food and other household expenses was common among the rural residents as on average they made nearly one type of trade-off in the past three months. The number of trade-offs was the highest among highly food insecure respondents (mean = 2.64), followed by moderately food insecure respondents (mean = 1.66); low food insecure respondents had the lowest number of trade-offs (mean = 0.39). The moderating effects of easiness in food access and dependence on food sources varied by level of food insecurity. The results show that individuals at different levels of food insecurity use different strategies to fulfill their food needs and social programs are more often utilized than personal food sources. We conclude with implications for addressing food insecurity in order to reduce the possibility of making trade-offs.

## 1. Introduction

Food insecurity has been defined as a condition when individuals “lack regular access to enough safe and nutritious food for normal growth and development and an active and healthy life” [1]. As such, food insecurity is both a cause and a symptom of other household challenges, including financial duress, housing insecurity, and increased health care costs. These challenges have especially intensified during the COVID-19 pandemic, as for many families, income streams were diminished, food sources were altered, health care challenges increased, and housing arrangements shifted to accommodate precautions necessitated by the pandemic [2,3,4]. To cope with these myriad challenges, individuals often engage in a variety of coping strategies. Making financial trade-offs is part and parcel of the cycle of poverty and experiences of food insecurity [5]. It is important to understand whether and how different levels of food insecurity may be related to making trade-offs, as food insecurity is different from other forms of household hardships and is more sensitive to changes in income [6].

### 1.1. Making Trade-Offs

Previous research reveals that food-insecure households are twice as likely to face competing demands and make trade-offs [7]. Calloway and colleagues [8] categorize such trade-offs as hunger-coping trade-offs (e.g., choosing between paying for food or paying for other household experiences), financial hunger-coping strategies (e.g., borrowing money, skipping bills, selling property), and rationing hunger-coping strategies (e.g., eating after children, eating less food). Low income, food-insecure households are more likely to make trade-offs between food and paying for medical bills as they are more likely to experience negative life events such as a major change in financial status, death of a spouse, losing a job and homelessness [9], which can lead to more health challenges and greater needs for medical spending. Family members may also limit food purchases to save money or reduce food intake to ensure other family members are fed at the cost of their own health [6]. In addition, low-income individuals are often led to make trade-offs between buying food or paying for household bills [10,11], including electricity [12]. Food budgets are often the first to be shortchanged in times of financial duress as families make trade-offs [13,14]. Researchers have documented the connection between both short-term and chronic, longer term food insecurity and housing insecurity [15,16].

### 1.2. Accessing Food through Informal and Formal Channels

In many cases, families rely on outside support, from family and friends or community organizations, to ensure their financial and food needs are met and to reduce the need to engage in trade-offs [17,18]. Smith et al. [19] showed that individuals relied on their informal networks to help moderate household food insecurity. Informal networks generally include close family members, but also can include neighbors, friends, store owners, and employers [20]. Networks not only provide food, but also information, transportation and other resources that expand access to food.

Formal support mechanisms include food pantries, churches, and other social service agencies [19]. Government support, such as through the Supplemental Nutrition Assistance Program (SNAP) or the National School Lunch Program, also ease families’ food and financial burdens. Research has demonstrated the impact of the availability of SNAP benefits for helping families forestall having to make trade-offs [21], as such benefits provide recipients with the “breathing room” to make choices that benefit their family, while still ensuring that all basic needs are met. Using food pantries to supplement food supplies is associated with less desirable scores on hunger-coping strategies, such as making trade-offs [8]. Long et al.’s [22] research showed that individuals who reported putting off buying medicine to pay for food used food pantries more often than those who did not put off buying medicine to pay for food.

Support networks are especially important for individuals living in rural areas [23,24]. Living in a rural community shapes the resources and support that families draw upon to navigate their experiences through a variety of challenges, including food insecurity [25]. The burden of transportation, low vehicle ownership, and distance to healthy, affordable food increases the burden on rural households and creates “inflexible tradeoffs with other household purchases, such as food” [26] (p. 87).

In South Carolina where the study participants reside, prevalence of household level food insecurity averaged at 12.6% in 2019–2021 which is above national average [27]. About 14.5% of the South Carolina population live in rural areas in 2020 and those living in rural South Carolina are socioeconomically disadvantaged compared to their urban counterparts. For example, in 2020, the average rural per capita income was $40,315 while the urban per capita income was $49,293. The poverty rate in rural South Carolina was 18.1%, compared with 13.1% in urban areas of the state. The unemployment rate in rural South Carolina was 6.8%, while in urban South Carolina, it was 5.9% [28].

For many families, the COVID-19 pandemic intensified existing financial challenges or introduced new financial challenges. Social distancing protocols, workplace and employment disruptions and supply chain challenges all impacted the quality and quantity of food available in households. The U.S. Department of Agriculture [29] reported that households experienced different challenges and engaged in diverse strategies (e.g., limiting food intake) during the pandemic, depending on the level of food insecurity experienced in the household (i.e., low food security vs. very low food security). Many families had to adjust spending to accommodate emerging and unexpected costs associated with the pandemic. In particular, the pandemic shaped strategies used by low-income and food insecure households for obtaining food, similar to the strategies taken in the aftermath of natural disasters, when obtaining food becomes a “complex puzzle” for families [30] (p. 2). Budgeting decisions and trade-offs likely took on different dimensions during the pandemic as families now had to consider the health risks associated with accessing food in the broader community.

### 1.3. Research Questions

In this paper, we focus on the trade-offs that individuals from rural South Carolina households in low-income areas made during the COVID-19 pandemic. Much prior research on this topic has focused on how trade-offs predict food insecurity [8,21] and other factors such as stress [31]. In this study, we instead examine how food insecurity predicts trade-offs to learn whether households at different levels of food insecurity engage in a different number of trade-offs. We also highlight the influences of food access and sources of food support on the relationship between food insecurity and the likelihood of making trade-offs. More specifically, we investigate the following research questions:

(1) Are there differences in the number of trade-offs made by households experiencing different levels of food insecurity (i.e., low, moderate or high food insecurity)?
(2) Are the differences in the number of trade-offs made by households experiencing different levels of food insecurity explained by dependence on various food sources and by the easiness of access to food?
(3) Are the differences in the number of trade-offs made by households experiencing different levels of food insecurity moderated by levels of dependence on various food sources and by the easiness of access to food?

The study results will be of interest to policy makers, funders and practitioners. Our focus on the predictors of trade-offs versus food insecurity, and differentiating between different levels of food insecurity, advances prior research. Learning more about how households navigate the complexities of food insecurity by making food trade-offs and relying on various types of support can lead to more carefully developed policies and programs for addressing food insecurity.

## 2. Materials and Methods

### 2.1. Sample

For this paper, we used data from a cross-sectional survey that is a part of a larger investigation of food insecurity in nine rural counties in South Carolina. The survey included questions about food insecurity, food access, physical activity, and several measures of perceptions of community well-being. Other survey items assessed community residents’ perceptions about how various dimensions of their lives had changed since COVID-19. Institutional Review Board approval was obtained from Clemson University IRB.

The study sample was a purposive, convenience sample of low-income, rural residents in nine rural South Carolina counties. To recruit survey participants, the study team leveraged campus-community relationships with organizations that provided services to this population during the pandemic. Partners included food pantries, senior centers, Cooperative Extension, faith organizations, and community centers.

Data collection occurred in two phases from August 2020 to July 2021. During the first phase (August 2020 to March 2021), data was collected by telephone. During the second phase (July 2021), we collected data in-person in four of our study counties. For the telephone survey, our community partners publicized our survey to the community through informational flyers and word-of-mouth and collected names and contact information of individuals who visited their agencies during the pandemic. For the in-person survey administration, we collected data at several community agencies in four of our study counties, including churches and food banks. Both telephone and in-person data collection were conducted by a trained team of students and staff from a public university. Participants had the option of completing the survey in either English or Spanish. Upon completion of the survey, respondents received a $10 gift card, via mail or in person. In-person surveys were completed in approximately 15 to 20 min on average.

Participant eligibility criteria included the following: (1) residing in one of the nine study counties and (2) being over the age of 18. For the telephone survey, the survey items were read to respondents over the phone and responses were recorded into Qualtrics™ data entry software. The data from the in-person surveys was entered into Qualtrics^TM^ upon completion of data collection. Across the nine counties, 1115 potential survey respondents were contacted, of whom 713 completed the survey, for a 64% response rate. In sum, 580 surveys were completed via telephone, and 133 were completed in person. After list-wise deletion of missing cases, this study included 650 respondents.

### 2.2. Measures of Hunger-Coping Trade-Offs

Similar to Calloway et al.’s [8] approach for studying hunger coping trade-off strategies, respondents were asked: “In the past 3 months, have you or anyone in your household had to choose between buying the food you need or paying for any of the following? Check all that apply.” The trade-offs included medicine or medicinal care; utilities (electricity or cell phone); rent or mortgage; gas or fuel for vehicle; other bills (childcare). The total possible count of trade-offs ranged from 0 to 5, with a higher score representing a greater frequency of facing decisions between paying for food or other household expenses.

### 2.3. Measure of Food Insecurity

The following five items from the USDA’s [32] U.S. Household Food Security Survey were used to assess food insecurity status in the three months prior to the survey:

- The food that I bought just didn’t last and I didn’t have money to get more. (Affirmative response = Sometimes or often true)
- I couldn’t afford to eat balanced meals. (Affirmative response = Sometimes or often true)
- Did you ever cut the size of your meals or skip meals because there wasn’t enough money for food? (Affirmative response = Yes)
- How often did this happen? (Affirmative response = almost every day or 2–3 days)
- In the last three months, did you ever eat less than you felt you should because there wasn’t enough money for food? (Affirmative response = Yes).

Based on the sum of affirmative responses to these five items, households were classified as experiencing low (0 or 1 affirmative response), moderate (2, 3 or 4 affirmative responses) or high food insecurity (5 affirmative responses). This categorization was similar to that used by the USDA in its definition of various levels of food insecurity [32].

### 2.4. Measures of Food Sources

Respondents were asked how often (on a 5-point scale, ranging from “never” to “every week”) their household had depended on each of the following food sources in the three months prior to the survey: food pantry; free meal (Salvation Army, community center); federal school lunch or breakfast program; hunting or fishing; friends, co-workers, or neighbors; relatives outside the home; community or personal garden, senior center food distribution programs. Based on factor analysis which showed that a three-factor solution explained 53% of the variances, we averaged answers on food pantry, free meal, and senior center food distribution programs to create an index for dependency on agency-provided food. We averaged answers on friends, co-workers, or neighbors and relatives outside the home to create an index for dependency on friends and relatives as a food source. We averaged answers on hunting or fishing and community or personal gardens to create an index for dependency on personal sources of food. Because the federal school lunch or breakfast program did not have a high loading on any of these factors, we included it as a separate source of food.

### 2.5. Measure of Food Access

Respondents were asked how easy it was to access (purchase or get) each of the following items in their local community: fresh fruits and vegetables; locally grown or home-made food items; food support services; farmer’s market or produce stands; and affordable food. The 3-point response options to each item included not easy, somewhat easy, and very easy. Because factor analysis showed that answers to these five items loaded on one factor which explains 44% of the variances, we averaged scores on these items to create an index for easiness of food access.

### 2.6. Sociodemographic Measures

Demographic variables included gender (male, female), age (18–44, 45–64, and 65+), race/ethnicity (non-Hispanic white, non-Hispanic Black, Hispanic, other races), marital status (married/partnered, widowed, divorced/separated, and never married), whether having children under age 18 in the household, employment status (employed, unemployed, retired, and unable to work), and family income (less than $20,000, $20,000–under $35,000, $35,000–under $50,000, $50,000–under $75,000, more than $75,000 and an missing income category if no family income was reported).

### 2.7. Analytic Strategy

We calculated descriptive statistics first for all respondents and then for respondents in each food insecurity category. Chi-square tests were used to examine whether the distribution of the study variables significantly differ among the three food insecurity groups. We then estimated three Ordinary Least Squares regression (OLS) models to examine the relationships of food insecurity, sources of food dependency, and easiness in food access with our measure of trade-offs between paying for food and other items. The first model included food insecurity and demographic controls to examine the association between food insecurity and trade-offs controlling for demographics variables. The second model added sources of food dependency and easiness in food access to examine how much of the association between food insecurity and trade-offs can be explained by sources of food dependency and easiness in food access. The third model added interaction terms between food insecurity and sources of food dependency and easiness in food access to examine more carefully whether the association between food insecurity and trade-offs was moderated by sources of food dependency and easiness in food access. All analysis were conducted with STATA [33].

## 3. Results

### 3.1. Descriptive Statistics

Descriptive statistics are represented in Table 1. The respondents were mostly female (83.9%). More than half of the respondents were non-Hispanic Black (55.1%). About 44% were married, 33% had kids under 18 in the household, 44% were employed, and 59% had family income less than $35,000. For the participants’ food insecurity status, 68.6% of respondents were categorized as experiencing low food insecurity, 20.7% moderate food insecurity, and 10.7% high food insecurity. On a scale from 1 to 5, the average levels of dependency on free food sources, friends/relatives, federal school food programs, and personal sources were 1.73, 1.58, 1.55, and 1.51 respectively. On a scale from 1 to 3, the average level of easiness in food access was 2.22.

In looking at the differences by level of food insecurity, the three food insecurity groups varied significantly in three sources of food dependency, and easiness in food access, with respondents experiencing a high level of food insecurity having the highest number of trade-offs, highest level of dependency on relatives/friends and dependency on federal school food programs, and the lowest level of easiness in food access. Respondents experiencing a moderate level of food insecurity experienced the highest level of dependency on food pantry/free meal/senior center. Respondents in the three food insecurity groups also significantly differed in all other sociodemographic characteristics except gender; those with high food insecurity tended to be younger, non-Hispanic Black, unmarried, had minor children in the household, were unemployed or not able to work, and had a lower family income.

Table 2 presents the number and types of trade-offs for all respondents by food insecurity status. On a scale from 0 to 5, the average number of trade-offs was 0.90. The average number of trade-offs was the highest among highly food insecure respondents (mean = 2.64), followed by moderately food insecure respondents (mean = 1.66), and low food insecure respondents had the lowest number of trade-offs (mean = 0.39). Of all five types of trade-offs, the prevalence of trade-offs was the highest among highly food insecure respondents (25.7–67.1%) and the lowest among low food insecure respondents (8.0–25.2%).

### 3.2. Regression Results

Results from the OLS regressions are reported in Table 3. The first model, which included food insecurity measures and sociodemographic variables, accounted for 35.4% of the variance in the number of trade-offs. After controlling for demographic characteristics, respondents who had moderate food insecurity had one more trade-off (b = 1.04) and respondents who had a high level of food insecurity had nearly two more trade-offs (b = 1.97), compared to those who had a low level of food insecurity (Model 1). These associations were slightly attenuated but remained strong and statistically significant after sources of food dependence and easiness in food access were added in Model 2. Such attenuation was mainly caused by easiness in food access, as such food access was negatively associated with trade-offs; that is, being one level higher on easiness in food access was associated with 0.38 points lower on the number of trade-offs. In addition, the level of dependency on federal school food programs was positively associated with trade-offs while other sources of food dependence were not significantly associated with trade-offs. The addition of sources of food dependence and easiness in food access accounted for an additional 2.4% of the variance in the number of trade-offs.

To examine whether sources of food dependence and easiness in food access moderate the relationship between food insecurity and making trade-offs, the interaction terms between food insecurity and sources of food dependence and easiness in food access were added in Model 3; these interaction terms significantly improved model fit as the R square increased by 2.6% to 40.4%. Among them, the interactions of moderate food insecurity with dependence on friends/relatives (b = −0.26) and easiness in food access (b = −0.51) were negative and significant or marginally significant. The interactions of high level of food insecurity with dependence on free food programs (b = −0.40), dependence on friends/relatives (b = −0.45), and easiness in food access (b = −0.59) were also negative and at least marginally significant. These results indicate that respondents with moderate or high levels of food insecurity made fewer trade-offs with increasing levels of dependence on free food programs and on friends/relatives, and they also made fewer trade-offs with increasing levels of easiness in food access. There is a positive interaction effect between moderate food insecurity and dependency on federal school food program (b = 0.18) which indicates that respondents with moderate food insecurity experienced more trade-offs with increasing dependency on federal school food programs.

## 4. Discussion

Food insecurity is a powerful predictor of a variety of social determinants of health and it shapes household coping strategies for addressing financial challenges. Families face complex choices in their attempts to ensure that their needs are met. This study showed that food insecurity is prevalent in rural South Carolina; nearly one-third of the respondents experienced food insecurity and one-tenth experienced a high level of food insecurity. Making trade-offs between paying for food and other household expenses is common among rural residents as on average they made nearly one type of trade-off in the past three months. There is a strong association between food insecurity and the number of trade-offs as those with higher levels of food insecurity made more trade-offs than their food secure counterparts. The relationship between levels of food insecurity and dependence on various sources of food was not always consistent (Table 1). Levels of food insecurity had a gradient association with dependence on friends/relatives, federal school food program, and easiness of food access. However, dependence on free food programs was highest among moderate food insecure respondents and there was no significant association between levels of food insecurity and dependence on personal food sources. These findings suggest that individuals at different levels of food insecurity use different strategies to fulfill their needs of food and social programs are more often utilized than personal food sources.

Consistent with previous research [8,11,12], this study found a strong relationship between food insecurity and making trade-offs, where individuals experiencing moderate or high food insecurity made more trade-offs than individuals experiencing low food insecurity. Our study, however, reveals a more nuanced picture. While levels of dependency on free food sources, friends and relatives and personal sources were not associated with making trade-offs, dependency on the federal school food program was. Higher levels of dependence on federal school food programs were associated with more trade-offs. This finding highlights that the relationship between food insecurity and federal sources of support and income is not straightforward. This is especially important to consider in the context of COVID-19. At the time of data collection, federally sponsored school food programs were made available to all children enrolled in public schools. All children were delivered meals at the time that students were home-bound, regardless of family income. Students that normally qualified for free and reduced-price meals additionally received SNAP emergency supplements during the pandemic. While resources such as school meals and increased SNAP allotments are important for many families, they are insufficient for reducing food insecurity. Future research could better deduce what other kinds of support, aside from free food, are needed to help to reduce the possibility of making trade-offs.

Food access partially explained the impact of food insecurity on making trade-offs, in that high food insecurity was associated with lower levels of easiness in food access, which in turn, led to more trade-offs. It may seem obvious, as shown in our study, that being able to access food through these various informal and formal food channels would decrease the likelihood of engaging in trade-offs, as obtaining food from these sources means that individuals can then use the freed-up money to pay other bills [19]. In other words, increased food access provides families with more control over other expenses, an important factor for coping and adapting to limited resources [9]. Our findings confirm recent research showing that social networks and community sources of food (i.e., congregate meal sites) are important for maintaining nutrition for rural residents [34].

Our study shows that such networks can moderate the influence of food insecurity on making tradeoffs. The buffering effects of dependence on friends and relatives on making trade-offs was especially strong for those experiencing moderate or high food insecurity. These individuals made fewer trade-offs, even if experiencing food insecurity. This finding is consistent with prior research indicating that strong social networks can mitigate the negative impact of food insecurity, as such support serves as a safety net when people must juggle bills to meet essential needs, including food, housing and health care [17,18,19].

Findings from this study highlight the importance for various stakeholders (e.g., health care providers, non-profit leaders, policy makers, etc.) to learn whether their clients are making these trade-offs and what is being sacrificed, and maybe optimized, in the process. These trade-offs, while a normal part of decision making in many households, should not be normalized [35]. It is essential to consider the broader food environment within which these decisions take place as there is a complex interplay between using formal and informal channels of support for addressing food insecurity and informal networks of support may help fill gaps in government support [20].

Several limitations of this study should be acknowledged. The cross-sectional survey did not allow us to examine changes across time. As our study is not longitudinal, it does not adequately capture the complexity of food insecurity over time, including the fact that families often have intermittent experiences with food insecurity [36] and have different experiences depending on their receiving government support or support from informal channels [8]. We did not examine the relationship between the different types of trade-offs, an important consideration for providing a more holistic perspective of the material hardships that households face [37]. For example, existing research indicates that food insecurity is positively associated with making trade-offs to pay utility bills [7]. Qualitative research, focused on the lived experiences of families facing multiple hardships can help to illuminate these complexities, such as the sequencing of using various strategies to address household hardships. For example, research has shown that rural families may rely on government resources only after they have pursued personal channels of support [38].

Our study did not investigate to what extent such trade-offs were a part of a broader set of strategies for addressing financial challenges and food insecurity and how the respondents themselves perceived these tradeoffs. A “next-generation of food (in)security research” views such trade-offs as indicative of a family’s resilience, rather than evidence of its shortcomings [9] (p. 342). Families make trade-offs in creative ways to not only make sure their immediate needs are met, but also as a strategy for demonstrating autonomy and empowerment within the context of a broader food system that disenfranchises them [20]. Future research needs to investigate the short-term and long-term impact of making trade-offs on food insecurity.

This anti-deficit perspective also recognizes that programs and policies cause and contribute to the intransigent cycle of poverty that leads to food insecurity and other household challenges. For example, individuals living in a state with higher rates of food insecurity used riskier strategies to address food insecurity (e.g., denying food for themselves), while individuals living in states with lower levels of food insecurity engaged their human capital in positive ways to ensure their food needs were met [39]. Research has established links between the trade-offs made at the federal level (e.g., policies regarding expanding or retracting SNAP benefits) and trade-offs at the household level (e.g., choosing to pay one bill over another) [21]. Thus, it is essential that policies are developed that mitigate the need to engage in such strategies by providing families with the financial stability required to ensure reliable access to healthy food. Unfortunately, this study was limited by a lack of data available on participation in SNAP and other federal food benefits programs.

## 5. Recommendations and Conclusions

This study fills a gap in the literature by providing a more in-depth analysis of the relationship between food insecurity and the number of trade-offs that families make. Further, this research is set within the context of rural South Carolina in Southeastern United States in and around the time of the COVID-19 pandemic. We highlight the nuanced and complex relationships between different types of hardships that households face when juggling competing demands on household finances. Our finding of a strong positive relationship between food insecurity and the number of tradeoffs highlights the need to develop social policies and programs to eliminate food insecurity. Our research also underscores the importance of food access, family and friends, and community agencies for mitigating the influence of food insecurity on making trade-offs. These findings call for the need of strengthening the food support system at various levels, including families, friends, community agencies, and federal and local governments, especially for those individuals experiencing food insecurity.

There are strategies and policies that could mitigate these challenges for rural residents. These include expanding federal supports for families during crises and emergency responses. SNAP, one of the most successful programs in the United States, according to the US Congressional Budget Office Center on Budget and Policy Priorities [40,41,42], has a long history of keeping families out of poverty. Disaster SNAP and PSNAP (Pandemic SNAP) have made a significant difference in people’s lives since these forms of supplemental food assistance help mitigate the trade-offs and decisions families make about rent, transportation, medical bills, and other daily needs [43]. A recent study [44] that examines longitudinal data over a 26-year period demonstrates that providing a social safety net for families through federal programming helps stabilize families and communities and has long term positive consequences and net benefits for society, i.e., social safety net programs work.

Further, accessing emergency food responses, and many food resources, requires strong community bonding, sharing of knowledge and social capital [45]. Day-to-day strategies that individuals and families employ to mitigate food and nutrition security require deep community ties and connections to others, shared knowledge about resources, and strategies at both the household level and the neighborhood and community level. Findings from this study suggest that SNAP expansion, stronger localized resource information banks, and greater bridging opportunities for individuals and communities to connect to resources are needed. The implications of these recommendations include not only federal and state policy expansion/adaptation of SNAP and other programs (e.g., expanded Women, Infant and Children (WIC), but also emphasis on regional and local planning to mitigate need in rural communities [46].

## Figures and Tables

**Table 1:** Descriptive statistics for all and by food insecurity status.

		Food Insecurity Status			
	All (*n* = 652)	Low (*n* = 447)	Moderate (*n* = 135)	High (*n* = 70)	*p* of Group Diff.
Female	83.9	83.5	82.2	90.0	
Age					***
18–44	29.0	27.7	24.4	45.7	
45–64	37.7	35.4	48.2	32.9	
65+	33.3	36.9	27.4	21.4	
Race/ethnicity					*
White, non-Hispanic	39.1	43.0	27.4	37.1	
Black, non-Hispanic	55.1	51.9	63.7	58.6	
Other race	3.4	3.1	5.9	0.0	
Hispanic	2.5	2.0	3.0	4.3	
Marital status					**
Married/partnered	44.3	48.6	38.5	28.6	
Widowed	13.5	12.1	13.3	22.9	
Divorced/separated	19.0	17.7	25.2	15.7	
Never married	23.2	21.7	23.0	32.9	
Children under 18 in HH	33.1	27.3	42.2	52.9	***
Employment status					***
Employed	43.6	46.1	38.5	37.1	
Unemployed	12.3	10.7	14.8	17.1	
Retired	32.2	35.6	28.9	17.1	
Unable to work	12.0	7.6	17.8	28.6	
Family income					***
Less than $20k	36.4	27.3	51.9	64.3	
$20k–under $35k	22.4	22.6	24.4	17.1	
$35k–under $50k	10.1	12.1	4.4	8.6	
$50k–under $75k	9.2	11.6	3.7	4.3	
$75k or more	11.2	15.2	3.0	1.4	
Missing	10.7	11.2	12.6	4.3	
Dependence on food pantry/free meal/sr. center (range: 1–5)	1.73 (0.81)	1.61 (0.78)	2.04 (0.86)	1.86 (0.73)	***
Dependence on friends/relatives (range: 1–5)	1.58 (0.87)	1.46 (0.77)	1.79 (1.04)	1.91 (0.96)	***
Dependence on federal school food program (range: 1–5)	1.55 (1.33)	1.45 (1.22)	1.49 (1.26)	2.33 (1.84)	***
Dependence on hunting/fishing/gardening (range: 1–5)	1.51 (0.90)	1.52 (0.91)	1.46 (0.82)	1.53 (0.94)	
Easiness in food access (range: 1–3)	2.22 (0.50)	2.29 (0.48)	2.08 (0.51)	2.00 (0.47)	***

Note: Numbers are mean (standard deviation) or percent; *** *p* < 0.001, ** *p* < 0.01, * *p* < 0.05 from chi-square or ANOVA tests.

**Table 2:** Number and type of trade-offs by food insecurity status.

		Food Insecurity Status			
	All (*n* = 652)	Low (*n* = 447)	Moderate (*n* = 135)	High (*n* = 70)	*p* of Group Diff.
Number of trade-offs (mean/std)	0.90 (1.43)	0.39 (0.95)	1.66 (1.56)	2.64 (1.71)	***
Trade-off for Medicine or medicinal care	20.3%	8.5%	38.5%	60.0%	***
Trade-off for utilities (electricity or cell phone)	25.2%	11.0%	50.4%	67.1%	***
Trade-off for rent or mortgage	17.2%	6.7%	32.6%	54.3%	***
Trade-off for gas or fuel for vehicle	19.2%	9.6%	31.1%	57.1%	***
Trade-off for other bills (childcare)	8.0%	3.6%	13.3%	25.7%	***

Note: Numbers are mean (standard deviation) or percent; *** *p* < 0.001 from chi-square or ANOVA tests.

**Table 3:** Unstandardized coefficients from OLS regressions on making trade-offs.

Variables	Model 1	Model 2	Model 3
Female	0.09	0.06	0.02
	(0.69)	(0.48)	(0.17)
Age (ref = 18–44)			
45–64	−0.05	−0.11	−0.12
	(0.36)	(0.89)	(0.93)
65+	−0.32	−0.43 *	−0.43 *
	(1.60)	(2.19)	(2.20)
Race/ethnicity (ref = White, non-Hispanic)			
Black, non-Hispanic	0.24 *	0.18 ^+^	0.19 ^+^
	(2.25)	(1.66)	(1.78)
Other race	0.15	0.14	0.20
	(0.54)	(0.54)	(0.75)
Hispanic	−0.48	−0.58 ^+^	−0.52 ^+^
	(1.57)	(1.91)	(1.73)
Marital status (ref = Married/partnered)			
Widowed	0.01	0.04	0.10
	(0.03)	(0.23)	(0.62)
Divorced/separated	0.10	0.05	0.03
	(0.75)	(0.38)	(0.23)
Never married	−0.03	−0.08	−0.08
	(0.26)	(0.67)	(0.67)
Children under 18 in household	0.34 **	0.21 ^+^	0.23 ^+^
	(3.02)	(1.70)	(1.94)
Employment status (ref = Employed)			
Unemployed	−0.05	−0.11	−0.04
	(0.31)	(0.75)	(0.26)
Retired	−0.06	−0.07	−0.03
	(0.36)	(0.40)	(0.20)
Unable to work	0.14	0.08	0.10
	(0.84)	(0.48)	(0.60)
Family income (ref = less than $20k)			
$20k–under $35k	0.02	0.02	−0.00
	(0.19)	(0.14)	(0.04)
$35k–under $50k	−0.36 *	−0.33 ^+^	−0.35 *
	(2.03)	(1.87)	(1.96)
$50k–under $75k	−0.28	−0.22	−0.22
	(1.52)	(1.19)	(1.15)
$75k or more	−0.41 *	−0.37 ^+^	−0.35 ^+^
	(2.20)	(1.95)	(1.84)
Missing	−0.25	−0.23	−0.24
	(1.53)	(1.39)	(1.48)
Food insecurity status (ref = Low)			
Moderate	1.04 **	0.96 **	2.08 **
	(8.53)	(7.70)	(3.41)
High	1.97 **	1.78 **	4.55 **
	(12.02)	(10.73)	(5.35)
Dependence on food pantry/free meal/senior center		0.08	0.13
		(1.28)	(1.62)
Dependence on friends/relatives		0.03	0.16 *
		(0.53)	(2.26)
Dependence on federal school food program		0.11 **	0.06
		(2.65)	(1.13)
Dependence on hunting/fishing/gardening		−0.04	−0.03
		(0.76)	(0.54)
Easiness in food access		−0.38 **	−0.19 ^+^
		(3.95)	(1.70)
Interactions of food insecurity status with food support and access			
Moderate insecurity × Dependence on agency sources			−0.01
			(0.11)
High insecurity × Dependence on agency sources			−0.40 ^+^
			(1.90)
Moderate insecurity × Dependence on friends/relatives			−0.26 *
			(2.12)
High insecurity × Dependence on friends/relatives			−0.45 **
			(2.65)
Moderate insecurity × Dependence on federal school food program			0.18 ^+^
			(1.91)
High insecurity × Dependence on federal school food program			0.03
			(0.38)
Moderate insecurity × Dependence on personal sources			0.10
			(0.71)
High insecurity × Dependence on personal sources			−0.03
			(0.20)
Moderate insecurity × Easiness in food access			−0.51 *
			(2.25)
High insecurity × Easiness in food access			−0.59 ^+^
			(1.85)
Constant	0.41 *	1.18 **	0.56
	(2.03)	(3.37)	(1.41)
R-squared	0.35	0.38	0.40

Note: t-statistics in parentheses; ** *p* < 0.01, * *p* < 0.05, ^+^ *p* < 0.1 (two-tailed tests).

## Data Availability

Due to privacy and ethical concerns, neither the data nor the source of the data can be made available.
